# Restricted valency (NPNA)_*n*_ repeats and junctional epitope-based circumsporozoite protein vaccines against *Plasmodium falciparum*

**DOI:** 10.1038/s41541-022-00430-y

**Published:** 2022-01-27

**Authors:** Mark D. Langowski, Farhat A. Khan, Sofya Savransky, Dallas R. Brown, Arasu Balasubramaniyam, William B. Harrison, Xiaoyan Zou, Zoltan Beck, Gary R. Matyas, Jason A. Regules, Robin Miller, Lorraine A. Soisson, Adrian H. Batchelor, Sheetij Dutta

**Affiliations:** 1 Structural Vaccinology Lab, Malaria Biologics Branch, Walter Reed Army Institute of Research, Silver Spring, MD USA; 2 Malaria Department, Naval Medical Research Center, Silver Spring, MD USA; 3 US Military HIV Research Program, Walter Reed Army Institute of Research, Silver Spring, MD USA; 4 Malaria Biologics Branch, Walter Reed Army Institute of Research, Silver Spring, MD USA; 5 United States Agency for International Development, Washington, DC USA; 6 Present Address: Pfizer, 401N Middletown Rd, Pearl River, NY 10965 USA

**Keywords:** Protein vaccines, Germinal centres

## Abstract

The Circumsporozoite Protein (CSP) of *Plasmodium falciparum* contains an N-terminal region, a conserved Region I (RI), a junctional region, 25–42 copies of major (NPNA) and minor repeats followed by a C-terminal domain. The recently approved malaria vaccine, RTS,S/AS01 contains NPNAx19 and the C-terminal region of CSP. The efficacy of RTS,S against natural infection is low and short-lived, and mapping epitopes of inhibitory monoclonal antibodies may allow for rational improvement of CSP vaccines. Tobacco Mosaic Virus (TMV) was used here to display the junctional epitope (mAb CIS43), Region I (mAb 5D5), NPNAx5, and NPNAx20 epitope of CSP (mAbs 317 and 580). Protection studies in mice revealed that Region I did not elicit protective antibodies, and polyclonal antibodies against the junctional epitope showed equivalent protection to NPNAx5. Combining the junctional and NPNAx5 epitopes reduced immunogenicity and efficacy, and increasing the repeat valency to NPNAx20 did not improve upon NPNAx5. TMV was confirmed as a versatile vaccine platform for displaying small epitopes defined by neutralizing mAbs. We show that polyclonal antibodies against engineered VLPs can recapitulate the binding specificity of the mAbs and immune-focusing by reducing the structural complexity of an epitope may be superior to immune-broadening as a vaccine design approach. Most importantly the junctional and restricted valency NPNA epitopes can be the basis for developing highly effective second-generation malaria vaccine candidates.

## Introduction

Malaria is a disease of significant public health importance and according to the WHO 2020 World Malaria Report, 409,000 deaths globally were attributed to malaria in 2019. Although there has been a decline in the number of cases and deaths since 2010, emerging drug and insecticide resistance threaten these successes. The spread of COVID-19 pandemic across Africa could further reverse decades of progress on malaria control as was noted during the 2014 Ebola virus outbreak in West Africa^1^. Of the five species of *Plasmodium* that can infect humans, *P. falciparum* (*Pf*) is the primary cause of malaria-associated mortality, with most deaths occurring in children under 5 years of age. The development of an effective vaccine would positively augment the fulfillment of 2040–2050 malaria eradication goals^2^.

When an infected *Anopheles* mosquito takes a blood meal *Plasmodium* sporozoites are injected in the skin and travel to the liver to establish infection. Sporozoites are covered with the Circumsporozoite protein (CSP) which has a N-terminal ‘domain’ encompassing Region I (RI), that includes an inter-species conserved 5 amino-acid motif (KLKQP), followed by a junctional sequence NP-DPNA-NPNV-DPNA, 25-42 copies of the major NPNA repeats, interspersed minor NPNV-DPNA repeat^3–5^, and a C-terminal region consisting of an alpha thrombospondin type-I repeat domain (Supplementary Fig. 1). *P. falciparum* strains show a high degree of conservation in the N-terminal and the repeat regions but the C-terminal region of CSP contains polymorphic residues surrounding a hydrophobic pocket^6^. CSP is the target of the most advanced malaria vaccine candidate, RTS,S/AS01 (Mosquirix, GSK Biologicals). The RTS,S/AS01 vaccine contains ~19 copies of the major NPNA repeats and a majority of the C-terminal region of CSP, linked to a Hepatitis B *S* antigen particle and is formulated in the adjuvant AS01^7^. RTS,S/AS01 received a positive scientific opinion by the European Medicines Agency^8^ and has recently been recommended by the WHO for use in children living in areas of moderate to high transmission of *P. falciparum* malaria. RTS,S efficacy in the field, measured as the incidence of first or only episode of clinical malaria, is estimated to be ~30%^9^. An improved form of RTS,S/AS01, which consists of R21 and Matrix-M adjuvant^10^, has also shown promise with >70% efficacy in an area of seasonal malaria transmission^11^. Additional efforts to improve the efficacy of CSP-based vaccines are currently underway^12^.

Structural studies of RTS,S-elicited monoclonal antibodies (mAbs 311 and 317) have shown that the major (NPNA)_n_ repeats, although flexible, frequently form short structured motifs: type-I beta and pseudo 3_10_ turns^13^. Stabilized by homotypic interactions several repeat-specific mAbs can bind to a single CSP molecule^14,15^. *Pf* sporozoite invasion inhibition (in vitro), as well as protection against transgenic parasites (in vivo), are primarily associated with mAbs binding to the central NPNA repeat region of CSP^13,16–23^. Similar to the emerging evidence from respiratory syncytial virus vaccine development, efforts to improve CSP vaccines involve the use of well defined protective epitopes identified using inhibitory mAbs^24–27^.

The structure-function relationship of CSP domains are not completely understood. The C-terminal region is anchored to the sporozoite via a GPI anchor and the rest of the molecule appears to have a flexible rod-like structure^28^. The RI and a putative proteolytic cleavage site within the N-terminal region play an important role in the process of hepatocyte invasion^3,29–31^. Mechanistically an N-terminal cleavage event is purported to convert CSP into an adhesive or invasive conformation^32–34^. Vaccine-elicited antibodies against cryptic N-terminal epitopes may block invasion^35^ and the presence of naturally-elicited N-terminal antibodies may be associated with protection^36^. Despite evidence of function, the N-terminal region was not included in the RTS,S vaccine construct and nearly full-length versions of CSP vaccines are now entering human clinical trials with the assumption that immune-broadening the response to the N-terminal region of CSP would improve vaccine efficacy^37–39^.

Whole sporozoite vaccines PfSPZ- or PfSPZ-CVac-elicited mAbs (e.g., CIS43, MGG4, 1210, 4493, and L9), along with some RTS,S-elicited mAbs (e.g., mAb 311) can bind to the junctional DPNA and NPNV motifs that adopt type I and pseudo 3_10_ turn conformations^16,17,21,22^. These junctional mAbs cross-react to varying degrees with the NPNA major repeats and initial indications show that vaccines that include the junctional region may be more effective than major repeat-based vaccines^23^. Multiple groups have tried to use this information in vaccine design: Tan et al. chemically conjugated the epitope for the junctional sequence cross-reactive mAb MGG4 (Table 1) to a carrier protein but failed to elicit anti-parasitic antibodies^17^, Jelinkova et al. chemically conjugated the epitope for the junctional mAb CIS43 to the Qß phage and demonstrated that functional and protective antibodies could be elicited in mice^40^ (Table 1). In another report, Francica et al. also showed that the junctional epitope displayed on a recombinant chikungunya VLP can produce protective antibodies in mice^24^. Antibodies that target adjacent regions such as murine mAb 5D5, which binds an N-terminal peptide EDNEKLRKPKHKKLK within the RI region (Table 1), was shown to block proteolytic processing of CSP and reduce liver parasite burden in a mouse passive transfer experiment^41^. Recent findings have also shown that a human IgA that targets the conserved RI sequence is inhibitory in a mouse model of infection^42^. While no potent mAbs against the C-terminal region has been reported^19^, broadening the responses to include epitopes in the N-terminal region in addition to the (NPNA)_*n*_ repeats has been hypothesized as means to improve CSP vaccines^16,17^.

**Table 1:** Amino acid sequences of antibody epitopes, vaccine constructs, and ELISA plate antigens used in this study.

Monoclonal antibody	Epitope sequence
CIS43	NPDPNANPNVDPNAN
MGG4	KQPADGNPDPNANPNVDPN
317	NPNANPNANPNA
580	NANPNANPNANPNANPNANP
**Vaccine construct**	**Immunogen sequence**
TMV-NPNAx5	(NPNA)_5_
TMV-Junc	NPDPNANPNVDPNANPNVDPNANPNA
TMV-Junc-NPNAx5	NPDPNANPNVDPNANPNVDPNA-(NPNA)_5_
TMV-RI	ENDDGNNEDNEKLRKPKHKKLKQPADG-NP-DPNA
TMV-NPNAx20	(NPNA)_20_
**ELISA plate antigen**	**Sequence**
FL-CSP protein	3D7 CSP containing 19 NANP & 3 NVDP
Repeat peptide	(NANP)_6_C
Junctional peptide	NPDPNANPNVDPNANPNVD

A problem with using short discrete epitopes as vaccines is that they are inherently weak immunogens. Virus-like particles (VLPs, 20–200 nm size range) are ideally sized for uptake by antigen-presenting cells and repetitive display of an epitope on VLPs further improves immunogenicity^43,44^. The tobacco mosaic virus (TMV) is being tested as an epitope display platform for developing vaccines against papillomavirus, influenza, tularemia, and plague^45–49^. TMV-based vaccines can be optimized using *E. coli* expression system and equivalent virions could also be produced *in planta*, to reduce manufacturing costs. Structurally, a TMV VLP is composed of 17 coat protein monomers that form a 20 nm disk. The disks can stack into rods up to ~300 nm in length allowing up ~2100 copies of an epitope to be displayed per TMV rod^50,51^.

Previously we used TMV to optimize the valency and flexibility of the (NPNA)_n_ repeat epitope^27^. Only five copies of NPNA (TMV-NPNAx5) induced higher antibody titers, avidity, and in vivo protection compared to a nearly full-length soluble CSP that contained 19 copies of NPNA per molecule. The observed superiority of TMV-NPNAx5 over full-length CSP was reproducible in the non-human primates where the TMV-NPNAx5 adjuvanted with ALFQ (the Army Liposome Formulation containing the QS-21) showed 3-fold improvement of repeat titers over the existing RTS,S/AS01 benchmark in humans. Here we used the TMV platform to test if vaccines based on the junctional sequence and the RI can compare to the TMV-NPNAx5 vaccine effectiveness. A TMV construct containing 20 copies of the NPNA repeat (TMV-NPNAx20) was also compared to TMV-NPNAx5. Our data support the continued development of CSP vaccines based on TMV VLPs and we show that epitope focusing by restricting (NPNA)_*n*_ valency or by displaying the junctional epitope can elicit equally potent protective antibodies.

## Results

### Design and production of TMV-Junc

**Fig. 1:**
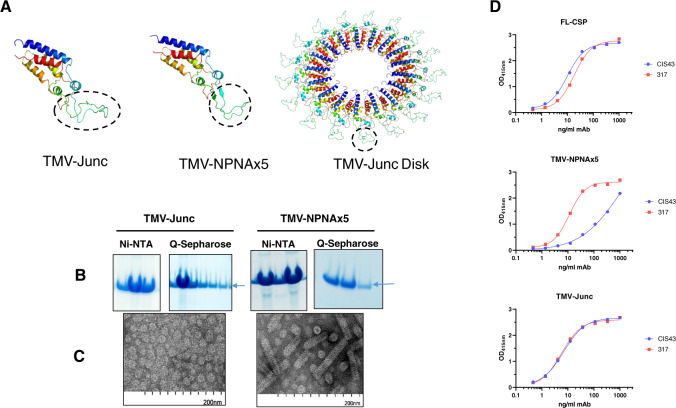
Design and characterization of TMV-based constructs used in Study 1. **A** Predicted monomer structures of circular permutants with inserted epitopes (circled). Left (TMV-Junc); middle (TMV-NPNAx5); and right (fully assembled TMV-Junc disk based on 3KML crystal structure). All structures were predicted by Rosetta Protein Prediction Server and images generated using PyMOL software. **B** Coomassie blue stained SDS-PAGE gels showing Ni-NTA and Q-Sepharose purification results for TMV-Junc and TMV-NPNAx5 proteins (arrow). **C** Electron micrographs of each purified vaccine preparation. **D** Representative ELISA titration curve of mAbs 317 (red) and CIS43 (blue) binding to FL-CSP, TMV-NPNAx5, and TMV-Junc (top to bottom).

Constructs containing CSP sequences were developed using a circular permutant of TMV as previously described^27^. The circular permutant sequence allows the N- and C-termini of the TMV coat protein to be engineered into the pore of an assembled disk, such that a peptide epitope can be displayed on the exposed loop^52^ (Fig. 1A). The 26 amino acids beginning at the junctional sequence NP-DPNA-NPNV-DPNA-NPNV-DPNA-NPNA were inserted into the exposed loop and the resulting construct was named TMV-Junc (Table 1). The comparator construct, TMV-NPNAx5, displayed five copies of the NPNA repeat on the exposed loop (Fig. 1A). The two antigens were expressed in *E. coli* as insoluble proteins and were purified under denaturing conditions by Ni-NTA and Q column chromatography (Fig. 1B). TMV-like disks and rods were confirmed by electron microscopy in both antigens (Fig. 1C). The average size as measured by dynamic light scattering (DLS) for TMV-Junc and TMV-NPNAx5 was 61.86 and 69.93 nm, respectively (Supplementary Fig. 2). TMV-NPNAx5, TMV-Junc, and a nearly full-length CSP antigen (FL-CSP) were tested for reactivity to mAbs 317 (preferential binding for NPNA) and CIS43 (cross-reactive binding to NPNA and junctional sequences). ELISA showed mAbs 317 and CIS43 bound to FL-CSP with similar efficiency; OD = 1 equivalent concentrations 10.9 and 6.0 ng/mL mAb respectively (Fig. 1D). In contrast, mAb 317 bound to TMV-NPNAx5 with 16-fold higher efficiency than mAb CIS43; 7.5 vs. 121 ng/mL respectively^27^. MAbs CIS43 and mAb 317 showed no difference in binding efficiency to TMV-Junc; 4 vs. 3.7 ng/mL respectively. These results confirmed the presence of the mAb CIS43 epitope on TMV-Junc_,_ and that this construct was antigenically distinct from TMV-NPNAx5.

### Study-1: TMV-NPNAx5 vs. TMV-Junc vs. FL-CSP

#### Immunogenicity

**Fig. 2:**
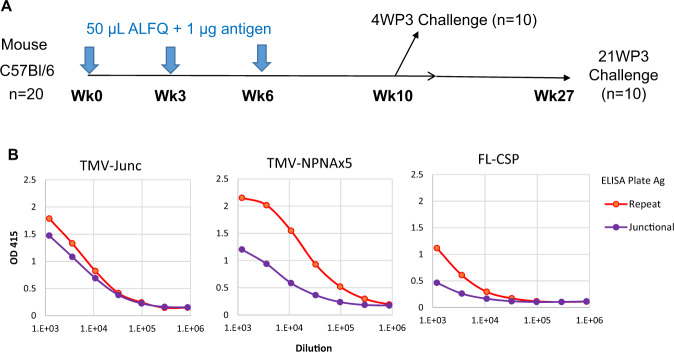
Study 1 design and immunogenicity for TMV-Junc_,_ TMV-NPNAx5, FL-CSP. **A** Outline of study design. **B** ELISA curves (mean of two experiments) showing reactivity to repeat (NANPx6) and Junctional peptide (KQPADGNPDPNANPNVDPN).

Three immunizations of 1 µg TMV-Junc, TMV-NPNAx5, or the FL-CSP antigens, formulated in 50 μL of adjuvant ALFQ^53^, were administered to C57BL/6 mice (*n* = 20) at week 0, 3, and 6 (Fig. 2A). A pool of sera from 2 weeks post 3rd dose (2WP3) showed that TMV-Junc antisera reacted similarly to the NANPx6 repeat peptide and to a junctional sequence containing peptide (KQPADGNPDPNANPNVDPN)^17^, while sera against TMV-NPNAx5 and FL-CSP vaccine groups preferentially bound to the repeat peptide over the same dilution range (Fig. 2B). Polyclonal antibodies to TMV-Junc, therefore, recapitulated the cross-reactivity of this antigen to mAbs 317 and CIS43 (Fig. 1D).

**Fig. 3:**
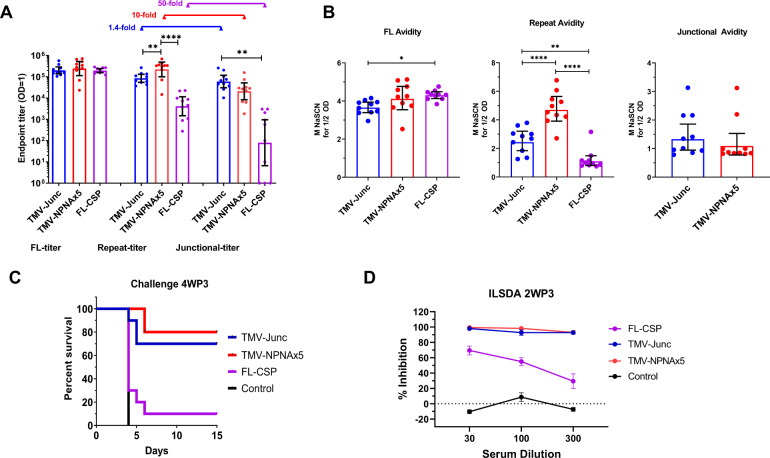
Study 1 immunogenicity and functional assessment of TMV-Junc, TMV-NPNAx5, FL-CSP in mice challenged at 4WP3. **A** Geometric mean ± 95% CI at 2WP3 against FL-CSP (FL), repeat peptide or junctional peptide and **B** Avidity at 2WP3 (defined as the molarity of NaSCN required to reduce OD to half maximal compared to PBS wash) against FL, NANPx6, and junctional peptide coat. Bars are geometric mean ± 95% CI. **C** Survival curves for mice challenged at 4WP3. **D** Mean ILSDA ± SEM from four experiments at 1:30, 1:100, and 1:300 using pooled serum dilutions from 2WP3. Inhibition was described as % reduction in 18s parasite RNA relative to the control wells. For ELISAs, statistical significance between groups was determined by one-way ANOVA followed by Tukey’s correction; **** (*p* < 0.0001), ** (*p* < 0.01), or * (*p* < 0.05).

ELISA against the FL-CSP plate antigen (FL-titer) on individual mouse sera at 2WP3 showed similar geometric mean titers in the three vaccine groups (Fig. 3A). The NANPx6 repeat titer was the highest for TMV-NPNAx5 group, being three-fold higher than TMV-Junc (*p* ≤ 0.01) and 23-fold higher than FL-CSP (*p* ≤ 0.001). The titer against the junctional peptide was highest for the TMV-Junc group, which was 2.3-fold higher than TMV-NPNAx5 (non-significant), and 90-fold higher than for the FL-CSP group (*p* ≤ 0.01) (Fig. 3A). The ratio of repeat:junctional peptide titer was 1.4 fold for TMV-Junc (Fig. 3A), while it was >10-fold for TMV-NPNAx5 and >50-fold for the FL-CSP group, further proving that TMV-Junc elicited polyclonal antibodies showed cross-reactive binding like mAb CIS43.

An avidity ELISA conducted using a chaotropic agent sodium thiocyanate (NaSCN), was used to show the relative strength of antibody binding (Fig. 3B). FL-CSP elicited antibody avidity for the FL-CSP plate antigen (FL-avidity) was not significantly different from that of TMV-NPNAx5 antibodies, but was higher than the avidity of TMV-Junc antibodies (*p* < 0.05). Repeat-avidity of the TMV-NPNAx5 group was higher than the other two groups (*p* values ≤ 0.0001), and TMV-Junc repeat-avidity was higher than FL-CSP group (*p* ≤ 0.01). Strikingly, junctional peptide avidity was low for the TMV-Junc and TMV-NPNAx5 induced antibodies (<1.5 M) and it was not detectable for the FL-CSP induced antibodies, suggesting that in general antibodies bound weakly to the junctional peptide in an ELISA, as compared to binding to the major repeat peptide.

#### Challenge at 4 weeks post 3rd immunization (4WP3)

Ten mice were challenged with 3000 transgenic sporozoites at 4WP3. All of the negative control mice became infected by day-4 while only 1/10 FL-CSP vaccinated mice exhibited sterile protection (Fig. 3C). Compared to the control group, the TMV-NPNAx5 (80%; Fisher’s exact test compared to controls, *p* = 0.0055) and TMV-Junc (70%; *p* = 0.01) elicited similarly high levels of sterile protection. In vitro liver stage sporozoite development assay (ILSDA) conducted using serum pools at 2WP3 for this group of challenged mice showed that TMV-Junc and TMV-NPNAx5 pools inhibited >90%, while FL-CSP pool consistently inhibited <70% at 1:30, 1:100 and 1:300 dilutions (Fig. 3D). The protection and ILSDA outcome in this experiment were similar to a previous experiment where 3 × 2.5 µg TMV-NPNAx5 and TMV-Junc antigens formulated in ALFQ were administered IM at week 0, 3, and 9 and the mice (*n* = 10) were challenged at 2WP3 with 3000 trangenic parasites (Supplementary Fig. 3).

#### Challenge at 21 weeks post 3rd immunization (21WP3)

**Fig. 4:**
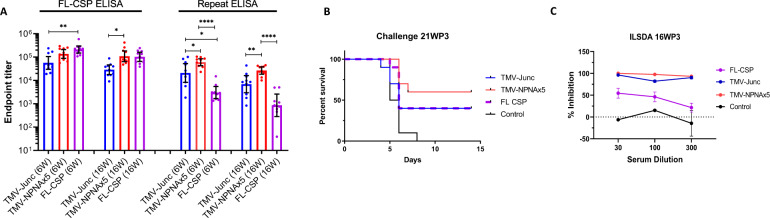
Study 1 immunogenicity and functional assessment at 21WP3 for TMV-Junc, TMV-NPNAx5, FL-CSP. **A** Geometric mean titer of serum at 6 and 16 weeks post 3rd (6 W, 16 W) for mice challenged at 21WP3 against FL-CSP (FL) and the repeat peptide. Bars are geometric mean ± 95% CI. Group means were compared by one-way ANOVA followed by Tukey’s correction; **** (*p* < 0.0001), ** (*p* < 0.01), or * (*p* < 0.05). **B** Survival curves of mice challenged at 21WP3. **C** Mean ILSDA performed at 1:30, 1:100, and 1:300 pooled serum dilution at 16WP3.

The 10 remaining mice in Study 1 were tested by ELISA at 6WP3 and 16WP3 to assess longevity (Fig. 4A). In this 10 week interval, the FL-titers dropped two-fold, 1.3-fold, and 2.1-fold and the repeat-titers dropped three-fold, 2.3-fold, and 3.5-fold in the TMV-Junc, TMV-NPNAx5, and FL-CSP groups respectively. Challenge of this group of mice 21WP3 with 3000 transgenic sporozoites showed 60% sterile protection in the TMV-NPNAx5 (Fisher’s exact test compared to controls, *p* = 0.0108) and 40% protection in the TMV-Junc and FL-CSP groups (n.s.) (Fig. 4B). ILSDA using serum collected at 16WP3 showed higher inhibitions for the TMV-NPNAx5 and TMV-Junc antibodies, consistent with the previous assay (Figs. 4C and 3D).

Study 1 demonstrated that the CSP junctional epitope (mAb CIS43 target) was weakly immunogenic on FL-CSP, and its display on TMV-Junc induced polyclonal antibodies that recapitulated the cross-reactive binding properties of mAb CIS43. TMV-NPNAx5 induced higher repeat-specific antibody titer than TMV-Junc and the TMV-NPNAx5 antigen induced similar levels of junctional epitope binding antibodies as TMV-Junc. Avidity data showed that the major NPNA repeat was the primary target of high-affinity binding of antibodies elicited by TMV-NPNAx5 and also TMV-Junc. The TMV-NPNAx5 and TMV-Junc antibodies had comparable in vitro and in vivo efficacy and were both superior to FL-CSP. The longevity of antibody responses did not differ between these vaccines.

#### Design and production of TMV-Junc-NPNAx5 and TMV-RI constructs

**Fig. 5:**
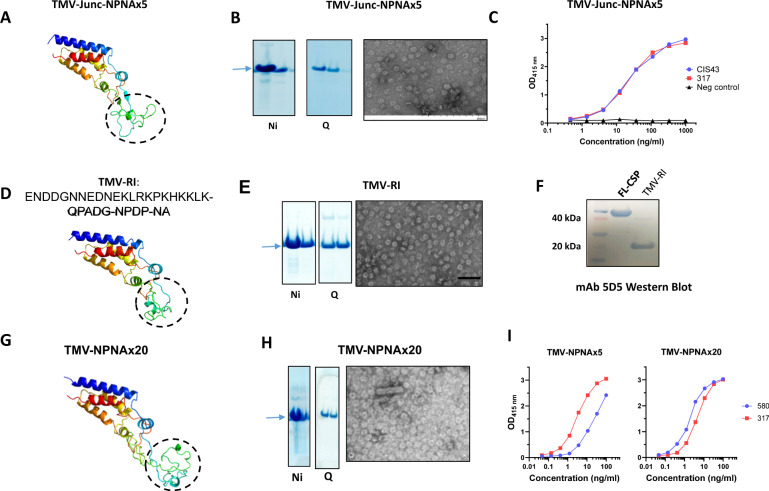
Characterization of antigens used in Study 2. TMV-Junc-NPNAx5, TMV-RI, and TMV-NPNAx20. **A**, **D**, **G** Rosetta predicted monomer structures of TMV-Junc-NPNAx5, TMV-RI, and TMV-NPNAx20. Images were generated using PyMOL. **B**, **E**, **H** Left panels show coomassie blue-stained SDS-PAGE gel bands (arrows) of Nickel and Q-Sepharose purified proteins; right panels show electron micrographs for each antigen. **C** ELISA reactivity curves of TMV-Junc-NPNAx5 binding to mAb CIS43, mAb 317, or PBS control. **F** Western blot using Region I specific mAb 5D5 against TMV-RI and FL-CSP antigen. **I** Representative ELISA curves of TMV-NPNAx20 and TMV-NPNAx5 reactivity to mAb 580 and mAb 317.

Protective responses induced by the junctional and the NPNAx5 epitopes prompted the design of a single TMV antigen displaying both epitopes. The sequence NP-DPNA-NPNV-DPNA-NPNV-DPNA-NPNAx5 was inserted at the exposed loop of TMV and the antigen was termed TMV-Junc-NPNAx5 (Fig. 5A). TMV-Junc-NPNAx5 was expressed, purified, and subsequent electron microscopy confirmed the presence of TMV-like disks (Fig. 5B). An ELISA showed that mAb 317 and mAb CIS43 bound to TMV-Junc-NPNAx5 with similar efficiency; OD = 1 equivalent concentration 8 and 9 ng/mL mAb respectively (Fig. 5C). Another TMV antigen, TMV-RI, was designed to display the RI and the mAb 5D5 epitope (Fig. 5D). The TMV-RI antigen was expressed, purified and electron microscopy showed TMV-like rods and disks (Fig. 5E). The TMV-RI and FL-CSP antigens reacted to mAb 5D5 as demonstrated by western blot (Fig. 5F). The average size determined by DLS for TMV-Junc-NPNAx5 and TMV-RI was 38.32 nm and 33.86 nm, respectively (Supplementary Fig. 2).

#### Design and production of TMV-NPNAx20

A TMV antigen containing 20 NPNA copies (TMV-NPNAx20), reported previously by Langowski et al.^27^, was also included in this study (Fig. 5G). TMV-NPNAx20 was expressed, purified, and electron microscopy confirmed the presence of TMV-like disk and rod forms (Fig. 5H). DLS measurement of TMV-NPNAx20 indicated an average particle size of 99.42 nm (Supplementary Fig. 2). TMV-NPNAx20 and the TMV-NPNAx5 reacted to two (NPNA)_*n*_ specific mAbs 580 and 317. MAb 580 is a low-affinity IgM isolated from a naturally exposed individual^18,23^ (Fig. 5I). MAb 317 is a high-affinity IgG isolated from a RTS,S vaccinee^13,23^. MAb 317 bound TMV-NPNAx5 with 10-fold higher efficiency than did mAb 580; OD = 1 equivalent concentrations 1.5 ng/mL *vs*. 12.2 ng/mL repectively (Fig. 5I). In contrast, mAb 317 bound the TMV-NPNAx5 and TMV-NPNAx20 with similar efficiency; 3.9 and 2.0 ng/mL respectively, thus confirming that restricting NPNA valency can specifically reduce the binding of a low-affinity mAb.

### Study-2: TMV-Junc-NPNAx5 vs. TMV-RI vs. TMV-NPNAx5 vs. TMV-NPNAx20

#### Immunogenicity

**Fig. 6:**
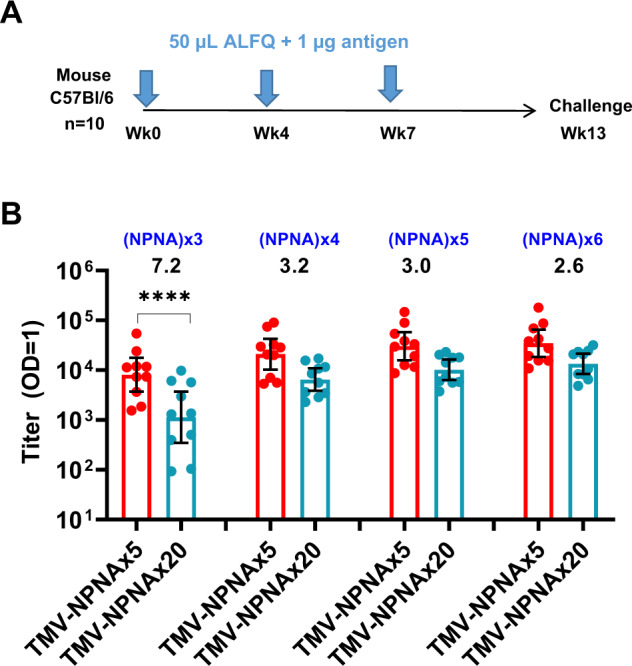
Study 2 study design comparing TMV-Junc-NPNAx5, TMV-NPNAx5, TMV-NPNAx20, TMV-RI, and repeat valency ELISA. **A** Outline of mouse study. **B** Geometric mean titer ± 95% CI for TMV-NPNAx5 and TMV-NPNAx20 sera against biotin-conjugated NPNAx3 to NPNAx6 peptides coated on avidin plates. The fold difference in titer between TMV-NPNAx5 and TMV-NPNAx20 and p values (Mann-Whitney Test, **** [*p* < 0.0001]) are shown.

Four groups of mice (*n* = 10) received three 1 µg doses of TMV-Junc-NPNAx5, TMV-RI, TMV-NPNAx5, or TMV-NPNAx20 vaccines formulated in 50 µL adjuvant ALFQ at 0–4 and 7-wk time-points (Fig. 6A). The timing of the third dose and the challenge were delayed due to COVID-19-related facility closures. Since the TMV-NPNAx20 and TMV-NPNAx5 differ only in the number of NPNA repeats, an ELISA against NPNAx1, x2, x3, x4, x5, and NPNAx6 peptides was performed on mouse sera from these two groups (Fig. 6B). No binding of polyclonal antibodies was observed to NPNAx1 or NPNAx2 plate antigens (not plotted). A trend towards increasing titer was observed as the repeat valency of the capture antigens increased. The TMV-NPNAx5 group titer was consistently higher than TMV-NPNAx20, but the difference in titer elicited by the 5 and 20 repeats decreased from 7.2-fold against NPNAx3 peptide (*p* < 0.0001) to 2.6-fold against NPNAx6 peptide (non-significant). Therefore restricted repeat valency in the vaccine preferentially improved titers to structurally constrained conformations of NPNA.

**Fig. 7:**
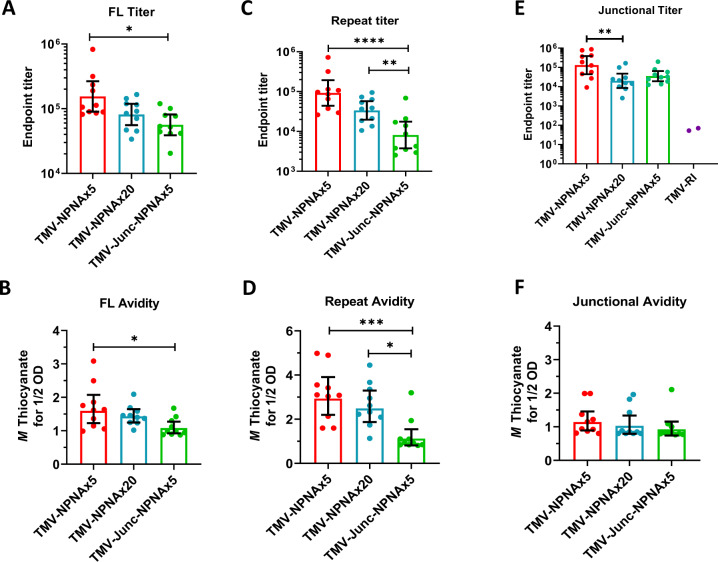
Study 2 geometric mean titers and avidity. At 5WP3 against FL-CSP (**A**, **B**); against NANPx6 (**C**, **D**); and junctional peptide (**E**, **F**). Bars are geometric mean ± 95% CI. Statistical significance between groups was determined by one-way ANOVA followed by Tukey’s correction; **** (*p* < 0.0001), *** (*p* < 0.001), ** (*p* < 0.01), or * (*p* < 0.05).

Five weeks post third dose (5WP3), the anti-FL titer of the TMV-NPNAx5 group was 2.3-fold higher than TMV-NPNAx20 (not significant) and 3.6-fold higher than TMV-Junc-NPNAx5 (p < 0.05). TMV-RI elicited no detectable FL-CSP specific responses (not plotted) (Fig. 7A). FL avidity of TMV-NPNAx5 was similar to TMV-NPNAx20 but it was significantly higher than the TMV-Junc-NPNAx5 (*p* < 0.05; Fig. 7B). Repeat titer for the TMV-NPNAx5 was three-fold higher than TMV-NPNAx20 (not significant) and ~10-fold higher than TMV-Junc-NPNAx5 (*p* < 0.05; Fig. 7C). The repeat titers of TMV-NPNAx5 (*p* < 0.0001) and TMV-NPNAx20 (*p* < 0.01) were both higher than TMV-Junc-NPNAx5 (Fig. 7C). The repeat avidity of TMV-NPNAx5 and TMV-NPNAx20 vaccines were similar and both were higher than TMV-Junc-NPNAx5 (*p* < 0.001 and *p* < 0.05, respectively, Fig. 7D). Junctional titer of TMV-NPNAx5 was seven-fold higher than TMV-NPNAx20 (*p* < 0.01) and was 5-fold higher than TMV-Junc-NPNAx5 (not significant) (Fig. 7E). TMV-RI group titers in most mice were undetectable against the junctional peptide that showed partial overlap with the RI sequence. Junctional epitope avidity was low for all vaccines (≤1 M NaSCN, Fig. 7F).

#### Functional analysis

**Fig. 8:**
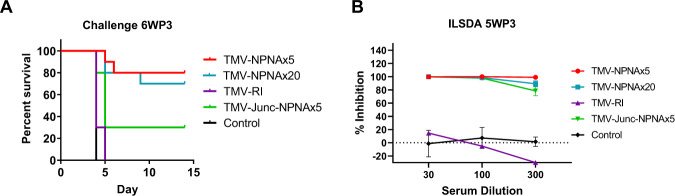
Study 2 functional assessment of TMV-Junc-NPNAx5, TMV-NPNAx5, TMV-NPNAx20, and TMV-RI. **A** Survival curves for parasite challenge at 6WP3. **B** ILSDA (mean ± SEM) on serum collected 5WP3 TMV-NPNAx5 (3 experiments) TMV-NPNAx20 (3 experiments), TMV-Junc-NPNAx5 (2 experiments), and TMV-RI (1 experiment) at 1:30, 1:100, and 1:300 dilution of serum.

Six weeks post third dose (6WP3) mice in Study 2 were challenged and all controls were infected by day 4 (Fig. 8A), but high level sterile protection was only observed in TMV-NPNAx5 (80%; *p* = 0.0007, relative to controls) and TMV-NPNAx20 (70%; *p* = 0.003) groups. Consistent with the lower titers, the protection achieved with TMV-Junc-NPNAx5 was lower (30%; not significant) and TMV-RI elicited no protection. ILSDA using serum pools at the 5WP3 showed that inhibition for TMV-NPNAx5 and TMV-NPNAx20 remained >90%; TMV-Junc-NPNAx5 inhibition dropped to 78% at the lowest serum dilution and TMV-RI induced no ILSDA activity (Fig. 8B).

Study-2 confirmed that epitopes for mAbs CIS43, 317, 580, and 5D5 epitopes could be recapitulated by TMV display and all except the mAb 5D5 epitope were displayed in immunologically favorable conformations. Restricting the repeat valency to NPNAx5 reduced the binding of a low-affinity mAb to this antigen and the resulting polyclonal antibodies to NPNAx5 reacted preferentially to shorter repeat peptides as compared to NPNAx20 antibodies. The TMV-NPNAx5 vaccine performance was as good if not better than TMV-NPNAx20. Most remarkably, combining the junctional epitope with NPNAx5 in one construct negatively impacted vaccine immunogenicity and efficacy. The RI region targeted by mAb 5D5 epitope was non-immunogenic even when displayed on the TMV particle and it induced no functional antibodies.

## Discussion

The use of ‘complete’ proteins or organisms as vaccines can potentially lead to co-induction of non-neutralizing antibodies that in extreme cases can exacerbate disease^54^. RTS,S/AS01 contains the NPNA repeats and the C-terminal region of CSP^55^. While the NPNA repeats are highly conserved, the C-terminal region of CSP is polymorphic^56^ and it induces strain-specific antibodies that may not be particularly inhibitory^19,57,58^ and lead to parasite escape^59–61^. A way to improve CSP vaccines is to eliminate non-neutralizing epitopes from the vaccine. We have shown that immune-focusing the antibodies to only the NPNAx5 epitope, elicited better protection than a nearly full-length recombinant CSP in mice and monkeys^27^. Here, epitopes of monoclonal antibodies that bind outside the (NPNA)_*n*_ repeats were tested for inhibitory effects and to determine if immune-broadening can improve upon TMV-NPNAx5 vaccine efficacy.

A region I construct (TMV-RI) did not induce CSP-reactive antibodies and our data did not support the inclusion of RI in a future vaccine. The high polarity and predicted flexibility of the RI sequence likely renders it non-immunogenic, even when stabilized as a loop on TMV. Despite being shown to be the target of an inhibitory mAb 5D5, RI-containing vaccines have not been highly protective without the repeat epitope^62^. A recent study using recombinant mAb 5D5 has also confirmed that this mAb may lack parasiticidal activity^63^.

The junctional epitope appears to be a highly susceptible target for inhibitory antibodies^16,17,22^, as evidenced by sterilizing protection observed in humans following mAb CIS43 passive transfer^64^. However, RTS,S (which lacks the junctional region) antibodies also bind to the junctional region^65^, and it has been suggested that junctional binding was a result of affinity maturation of (NPNA)_*n*_-specific antibodies^21,66^. To our knowledge, there has been no direct comparison of vaccines displaying the junctional and the major repeat region of CSP. Our TMV-Junc vaccine performed, as well as the TMV-NPNAx5 vaccine in a mouse model (Fig. 3). This was a remarkable result because TMV-Junc antibodies had low avidity for the junctional peptide and the repeat titer and avidity of TMV-Junc antibodies was also lower than that elicited by TMV-NPNAx5, these data suggested that polyclonal junctional antibodies may possess additional biological activities as observed for junctional mAbs^22^. While TMV-Junc antibodies recapitulated the binding properties of mAb CIS43, further studies are needed to distinguish the mode of parasite inhibition of TMV-Junc and TMV-NPNAx5 antibodies. Truncation of the TMV-Junc epitope or including the adjacent species-conserved KLKQPADG residues may achieve higher junctional specificity. Additionally, priming with TMV-NPNAx5 and boosting with TMV-Junc may be the most optimal conditions to recapitulate the maturation pathway and evolution of cross-reactive antibodies^21^.

Having established the protective efficacy of the junctional and (NPNA)_*n*_ epitope, we tested if immune broadening approach using epitopes for mAb CIS43 and 317 on the same construct can enhance efficacy. While the TMV-Junc was not included in Study 2, the TMV-Junc-NPNAx5 titer, avidity, ILSDA and protection were less than TMV-NPNAx5 (Fig. 5). This result was intriguing because fusion of NPNAx5 to the Junctional epitope reduced the immunogenicity of the entire construct. This result may be due to reduced stability of the TMV scaffold or destruction of a specific conformational epitope when junctional sequence was fused to NPNAx5. Alternatively, the cross-reactive junctional region antibodies may be masking the NPNAx5 epitope and vice versa, thereby reducing the overall immunogenicity of the construct^67,68^. The reduced protection for TMV-Junc-NPNAx5 vaccine echos a similar efficacy result where a truncated CSP with 9 major repeats showed better protection than a CSP of the same length with interspersed minor repeats^69^. We may therefore need to optimize cadence and epitope context for presenting these two short epitopes in tandem^70^. At the parasite level, Vijayan et al.^71^ have shown possible interference when monoclonal antibodies to the major and minor repeats of *P. yoelii* CSP are bound simultaneously; likewise, there is no evidence that *P. falciparum* mAb 317 and CIS43 can synergize in vitro (unpublished data). Therefore, immune-broadening using multiple short inhibitory epitopes on a single vaccine construct was complex and, in this instance, was found detrimental to vaccine efficacy.

While no evidence favoring epitope broadening with TMV-Junc-NPNAx5 was found, immune focusing by restricting repeat valency to NPNAx5 showed >80% sterile protection in two challenge experiments with 3 × 1 μg antigen dose and increasing the repeat valency to NPNAx20 did not further improve efficacy (Fig. 8). The efficient binding of mAb 317 to TMV-NPNAx5, but not the low affinity mAb 580, suggested that antibodies elicited by TMV-NPNAx5 may be qualitatively different from TMV-NPNAx20 antibodies. The low-affinity mAb 580 recognizes an extended (NPNA)_*n*_ conformation, which has been shown as being potentially less protective as compared to high-affinity antibodies like mAb 317, which recognizes a “curved” conformation^23,72^. Thus TMV-NPNAx5 immunogen may represent a significant reduction in potentially sub-optimal conformations of (NPNA)_*n*_ while still retaining the high-affinity conformations. We speculate that long repeat lengths present on parasite CSP function as a smoke-screen to allow the induction of non-effective low-affinity antibodies that may compete with high-affinity functional antibodies. Seder and Cockburn, have suggested that long CSP repeats could diminish the quality of CSP-specific B cells by allowing the formation of multivalent complexes with low-affinity B cells which then prematurely exit from germinal centers^73^. Our data shows that immune-focusing by restricting repeat valency may be a way to improve upon the current long-repeat containing CSP vaccine candidates.

TMV-Junc contained six full tetrapeptide repeats including one NPNA, three DPNA and two NPNV motifs^13,22^. This immunogen reacted positively to two potent inhibitory mAbs 317 and CIS43 and its insert encompassed NA-NPNV-DPNA-NPNV-D shown to be targeted by another highly effective mAb L9^22^. Others have also shown that junctional peptides fused to a VLP or as peptide immunogen can elicit functional antibodies^24,40,70^. While only a head to head comparison of vaccine platforms can be definitive, chemical conjugation of FL-CSP antigen to the Qß VLP did not improve upon the efficacy of an adjuvanted soluble protein FL-CSP^74^. In contrast, TMV-based VLPs reproducibly elicited higher antibody titers, avidity, and functional antibodies compared to similarly adjuvanted soluble CSP^27^, thus proving TMV to be a highly versatile platform for vaccine antigen display.

We assessed multiple CSP vaccines using a transgenic mouse parasite challenge model and an in vitro assay ILSDA that used *P. falciparum* parasites and human hepatocytes. A major caveat of our approach is that although the antibody titers have been associated with RTS,S mediated protection^75^, ILSDA, and mouse protection by intravenous challenge, are not proven correlates of human protection. Furthermore, mice do not harbor homologs to IGHV3-33/30 genes that are frequently elicited by the most potent human antibodies^20,21^ and we have previously reported that there is a disconnect between in vitro and in vivo mouse challenge readouts, particularly for junctional mAbs^23^. Overall, these data suggest that TMV-based CSP VLPs combined with the adjuvant ALFQ are excellent second-generation malaria vaccine candidates.

## Methods

### TMV structure prediction

The TMV-NPNAx5, TMV-Junc, and TMV-Junc-NPNAx5 structural models were submitted to the Rosetta Protein Structure Prediction Server (http://robetta.bakerlab.org)^76–78^. The predicted molecular model figures were generated using PyMOL software (Schrodinger, New York, NY).

### Production and purification of TMV-CSP particles

CSP epitope-fused TMV particles were generated by expressing fusion proterins in *E. coli*^27^. The genes were commercially optimized and synthesized for high level expression in *E. coli*, cloned into a inducible expression plasmid, and transformed into BL21 (DE3) cells. Cells were grown in shake flasks, induced with 0.1 mM IPTG at 37 °C for 2 h, and harvested by centrifugation. The insoluble cell pellet obtained after microfluidization and centrifugation was dissolved in 7 M urea containing buffer and purified using Qiagen Ni-NTA Sepharose (Gaithesburg MD) for the removal of non-specific proteins followed by Q-sepharose column for the removal of endotoxin (GE Lifesciences) under denatured conditions. The Q-sepharose flow-through was dialyzed overnight against a 20 mM Tris, 20 mM sodium chloride buffer (pH 9.0) containing 0.05% β-mercaptoethanol. The dialysis buffer was exchanged with fresh 20 mM sodium phosphate dibasic, 20 mM sodium chloride buffer (pH 7.4). After dialysis, the proteins were filtered using a 0.22 µm filter and concentrated on an Amicon ultra centrifugation filter (MilliporeSigma, Burlington, MA). Purity was of the final products was >95% as confirmed by analyzing the coomassie blue-stained SDS-PAGE gels (Supplementary Fig. 4). Protein constructs were stored at −80 °C before vaccination.

### Negative stain electron microscopy

TMV constructs were diluted in 20 mM sodium phosphate dibasic, 20 mM sodium chloride pH 7.4. 3 µL of diluted TMV sample was then applied onto Carbon-Formvar 300 Mesh copper grids (EM sciences) for 5 min and excess liquid was blotted away with bibulous paper. Grids were then stained with 3 µL of 1–2% uranyl acetate for 10 s before wicking of excess liquid with bibulous paper. The grids were imaged between 30,000X and 40,000X.

### Antigenicity ELISA

Human monoclonal antibodies 317, 580, and CIS43 were recombinantly produced^23,27^. Serial dilutions of mAbs were tested and the concentration that resulted in OD_415nm_ = 1 was determined using the Full-length CSP, TMV-Junc, TMV-NPNAx5, and TMV-Junc-NPNAx5 coated ELISA plates (100 ng protein-coated/well). ELISA plates were washed three times with 0.05% PBS-Tween solution, blocked for 1.5 h with blocking buffer (0.5% casein + 1% Tween) and mAbs were incubated for 1 h, starting at 1000-100 ng/mL concentration and serially diluted three-fold. A 1:4000 dilution of Goat anti-human HRP conjugated antibody (Southern Biotech, Birmingham AL; Cat# 2040-05) was applied to the wells and the plate was developed using ABTS Peroxidase Substrate (KPL Seracare Milford MA)^53^.

### Western blot

Mouse mAb 5D5 was kindly provided by Leidos Health (Reston, VA). A western blot was conducted using 1 μg protein separated by reducing PAGE and transferred to PVDF membrane. Immune-blots were stained with 1 μg/mL mouse mAb 5D5, followed by 1:5000 anti-mouse HRP conjugated antibody (Southern Biotech) and the blot was developed for 2 min using the Blue POD Substrate (Roche)^79^.

### Mouse serum immunogenicity ELISA

Mouse serum antibody titers against the junctional peptide (KQPADGNPDPNANPNVDPN)^17^ were determined following overnight coating at 100 ng/well; the NANPx_6_ peptide was coated at 20 ng/well, and the FL-CSP was coated at 100 ng/well. The remaining ELISA protocol and wash steps were similar to the antigenicity ELISA and secondary antibody was 1:4000 Goat anti-mouse H + L HRP (Southern Biotech; Cat#1036-05). Titer was defined as the serum dilution that resulted in OD_415_ = 1^53^.

### Mouse repeat valency ELISA

ELISA plates were coated with Avidin (100 ng/well) followed by 100 ng/well N-terminally biotin tagged peptides NPNAx1 to NPNAx6 (GenScript Piscataway, NJ). The remaining mouse serum ELISA was as described above.

### Thiocyanate avidity ELISA

A sodium thiocyanate ELISA was used to determine serum avidity^79^. ELISA plates coated with FL-CSP protein, NANPx6 or the junctional peptide were blocked with 200 µL of 0.5% Casein-PBS for 2 h at room temperature. Following a PBS-Tween wash, 100 µL of 1:1000 diluted serum were plated for 1 h at room temperature. Unbound antibodies were washed with 100 µL of aqueous NaSCN solution (0 M, 1 M, 2 M, 3 M, 4 M, 5 M, or 6 M) for 15 min at room temperature. After washing, the mouse serum ELISA was developed as above. To determine avidity index, the PBS washed well OD_415_ was used as the reference maximal, and linear regression was used to determine the concentration of NaSCN that reduced the OD_415nm_ to half maximal OD_415_.

### Inhibition of liver stage development assay (ILSDA)

ILSDA was performed using NF54 *Pf* parasites incubated with pooled sera from naïve or immunized mice at 1:30, 1:100, and 1:300 dilution or a positive control anti-CSP monoclonal NFS1^80^. This mixture was then added to wells containing human hepatocytes and after 3 days of incubation, the levels of *Pf* 18S rRNA levels were determined by quantitative real-time PCR. Percent inhibition was calculated against the naïve serum negative control and the assay was repeated more than once. All assay results were replicated in at least two independent experiments.

### Antigen formulations

All the TMV vaccine candidates tested were solubilized in 20 mM sodium phosphate dibasic, 20 mM sodium chloride (pH 7.4). Antigens were produced and vaccinations were conducted at different times. The antigens were also co-analyzed using SDS-PAGE, Electron Microscopy, and particle Malvern Zetasizer Nano S particle sizer (Supplementary Fig. 2). Particle size and the polydispersity indicies were calculated using Malvern Panalytical Software as average of 3 runs.

### Immunizations

The animal work was performed at the Walter Reed Army Institute of Research, Silver Spring Maryland USA under the mouse protocol #19-MVD-15, reviewed and approved by the Walter Reed Army Institute of Research Institutional Animal Care and Use Committee (IACUC). Animals were housed in a AAALACi accredited facility in compliance with the Animal Welfare Act and other federal statutes and regulations relating to animals and experiments involving animals and adheres to principles stated in the Guide for the Care and Use of Laboratory Animals, NRC Publication, 2011 edition.

All antigens were formulated in Army Liposome Formulation (ALFQ) that contains immune-modulators (3D-PHAD^TM^ and QS21)^81,82^. For Study 1, female C57BL/6 mice were vaccinated with 1 µg of either TMV-NPNAx5, TMV-Junc, or FL-CSP in ALFQ or sterile 1×PBS (control) at 0, 3, and 6 weeks. For Study 2, mice were vaccinated with 1 µg of either TMV-NPNAx5, TMV-NPNAx20, TMV-Junc-NPNAx5, or TMV-RI at 0, 3, and 7 weeks. All vaccines were 50 μl formulations administered intramuscularly (IM) in alternating thighs.

### Transgenic malaria challenge

Mice were challenged following the third dose with *P. berghei* expressing full-length *P. falciparum* CSP transgenic sporozoites^83^. Sporozoites were isolated from salivary glands of 18–22 days old mosquitoes via the Ozaki method, and maintained on ice until use within two hours. The sporozoites were suspended in GIBCO/Invitrogen RPMI 1640 Medium (1×) containing 5% fresh C57BL/6 mouse serum. Mice were challenged with 3,000 sporozoites per mouse in 100 µL volume injected intravenously (IV) into one of the lateral caudal veins of the tail with a 27G needle insulin syringe^57^. Mice were evaluated for parasitemia beginning at day 3 post-challenge through day 14 via Giemsa-stained thin blood smears. Thin blood smear slides were prepared by snipping the distal end of the tail and spotting a drop of blood onto a microscope slide. Smears were fixed with 100% methanol and stained with a 1:15 Giemsa in staining buffer solution for 30 min at room temperature. The slides were then rinsed with water and submerged in staining buffer for 1 min. After air drying, the slides were read using 100× oil immersion microcopy. Mice were euthanized with carbon dioxide, followed by cervical dislocation after showing positive blood smears for two consecutive days. Mice that remained non-parasitemic for 14 days post challenge were considered as protected and any mouse that was slide positive on two consecutive days was sacrificed and considered non-protected.

### Statistical analysis

Statistical significance for ELISA data was calculated by ANOVA and *p*-values were corrected for multiple comparisons using the Tukey’s method. Statistically significant differences in group means was indicated in figures as: **** (*p* < 0.0001), *** (*p* < 0.001), ** (*p* < 0.01), or * (*p* < 0.05). Protection outcomes were compared using Fisher’s Exact Test between groups. All statistical analyses and graphing were performed using GraphPad Prism Software (La Jolla, CA).

### Reporting summary

Further information on research design is available in the Nature Research Reporting Summary linked to this article.

## Supplementary information

Supplementary Material

REPORTING SUMMARY

## Data Availability

The datasets generated during and/or analyzed during the current study are available from the corresponding author on reasonable request.
