# Supplementary material for: Restricted valency (NPNA)n repeats and junctional epitope-based circumsporozoite protein vaccines against Plasmodium falciparum
Source: NPJ Vaccines. 2022 Jan 27;7:13. doi: 10.1038/s41541-022-00430-y (PMC8795123; doi:10.1038/s41541-022-00430-y)
Supplement: Supplementary file 1 — Supplementary Material [file 41541_2022_430_MOESM1_ESM.pdf]

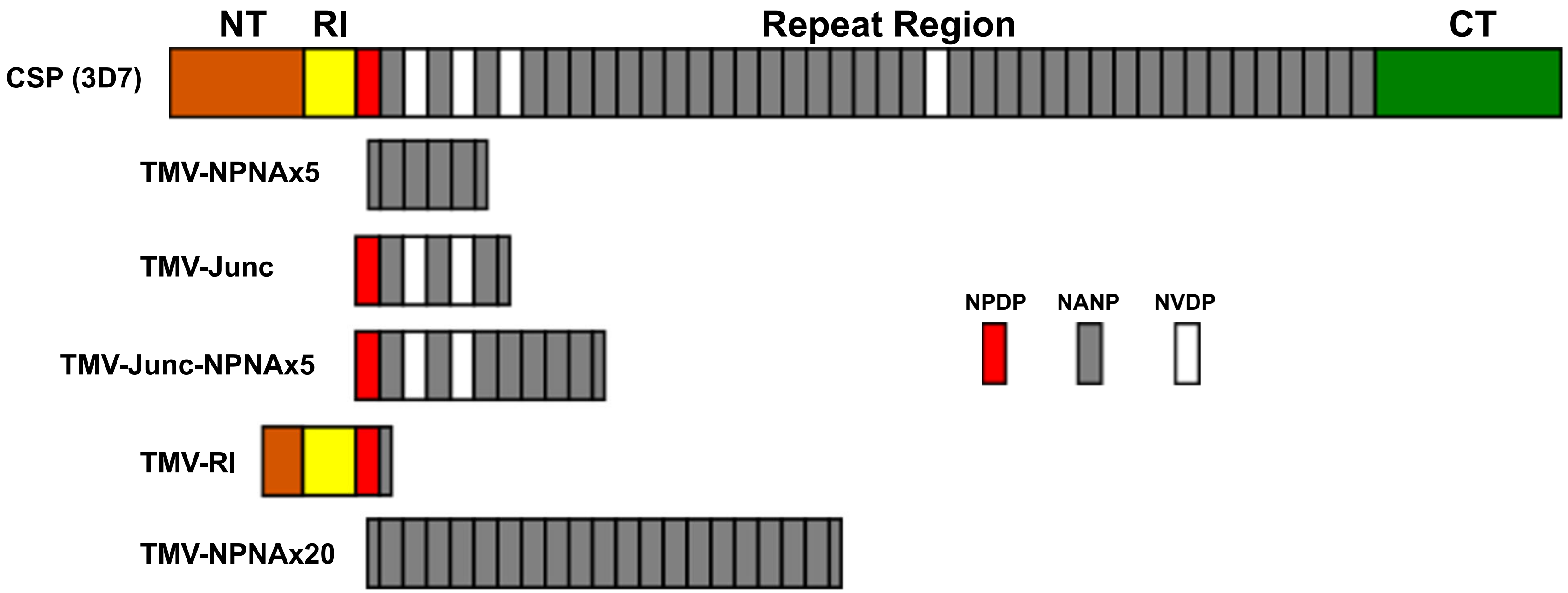

Supplementary Figure 1. Cartoon representation of the 3D7 strain of CSP (not to scale) and the immunogens used in the study. NT = N-terminal region, RI = Region I, and CT = C-terminal region. The junctional sequence is represented in red (NPDP), the major repeat in dark gray (NANP), and the minor repeat in white (NVDP). Repeat boxes that are half the size represent half the sequence in the proper cadence. TMV-RI encompasses mAb 5D5 epitope which contains Region I and its upstream sequence. Graphic was generated using Inkscape.

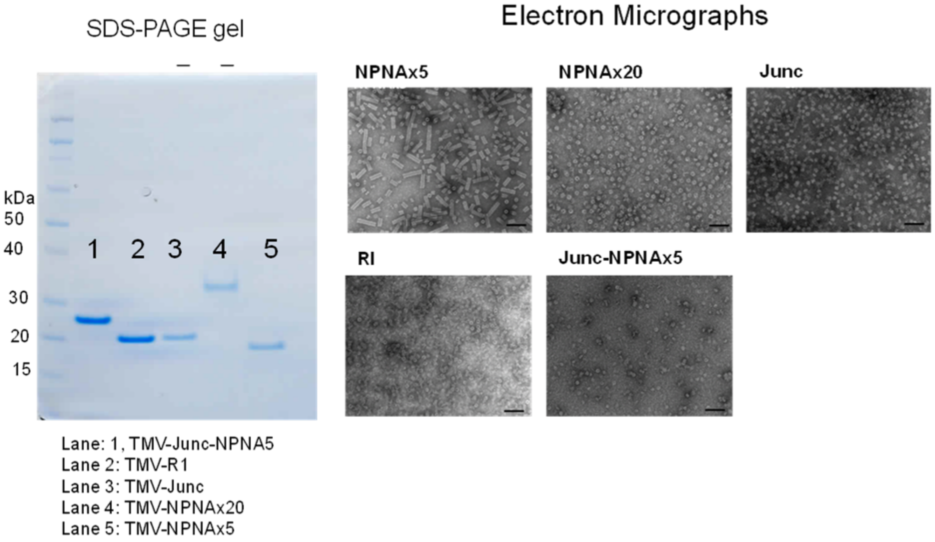

Dynamic Light Scattering

Vaccine	Z-Average (d.nm)	Peak 1 (d.nm, %)	Peak 2 (d.nm, %)	PdI (Polydispersity Index)
NPNAx5	69.93	80.07, 93.1%	2737, 6.9%	0.252
NPNAx20	99.42	177.0, 85%	31.85, 15%	0.412
Junc	61.86	186.3, 67.2%	25.70, 32.8%	0.553
RI	33.86	37.72, 96.3%	4778, 3.7%	0.245
Junc-NPNAx5	38.32	66.94, 100%		0.277

Supplementary Figure 2. Summary stability data for immunogens used in this study. All proteins used in the vaccine study were thawed and analyzed by coomassie blue staining of SDS-PAGE gel, negative-stain electron microscopy (bar represents 100 nm) and particle size distribution by dynamic light scattering (intensity).

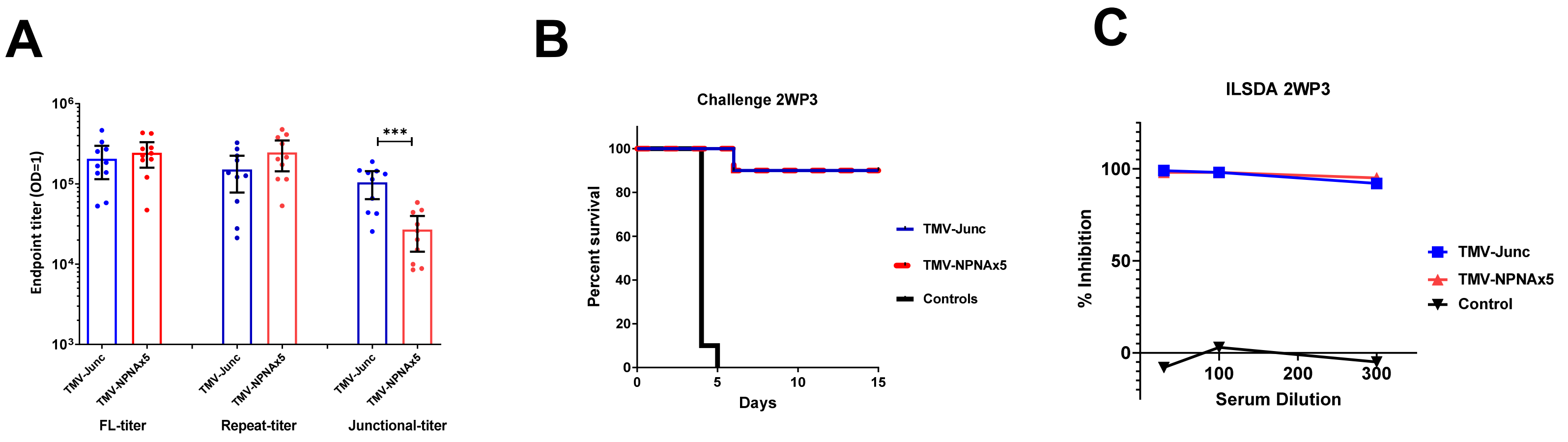

Supplementary Figure 3. Immunogenicity and functional assessment of TMV-Junc *vs.* TMV-NPNAx5. 2.5 µg antigen formulated in ALFQ was administered IM in C57Bl/6 mice (n=10) at week 0-3 and 9 (delayed 3rd dose). Groups were compared by 2-tailed T-test; *** ($p < 0.001$). **(A)** FL-titer, repeat-titer and junctional-titer of serum at 2WP3. **(B)** Survival of mice following challenge with 3000 transgenic *P. berghei* parasites at 2WP3 **(C)** Mean ILSDA performed at 1:30, 1:100, and 1:300 pooled sera at 2WP3.

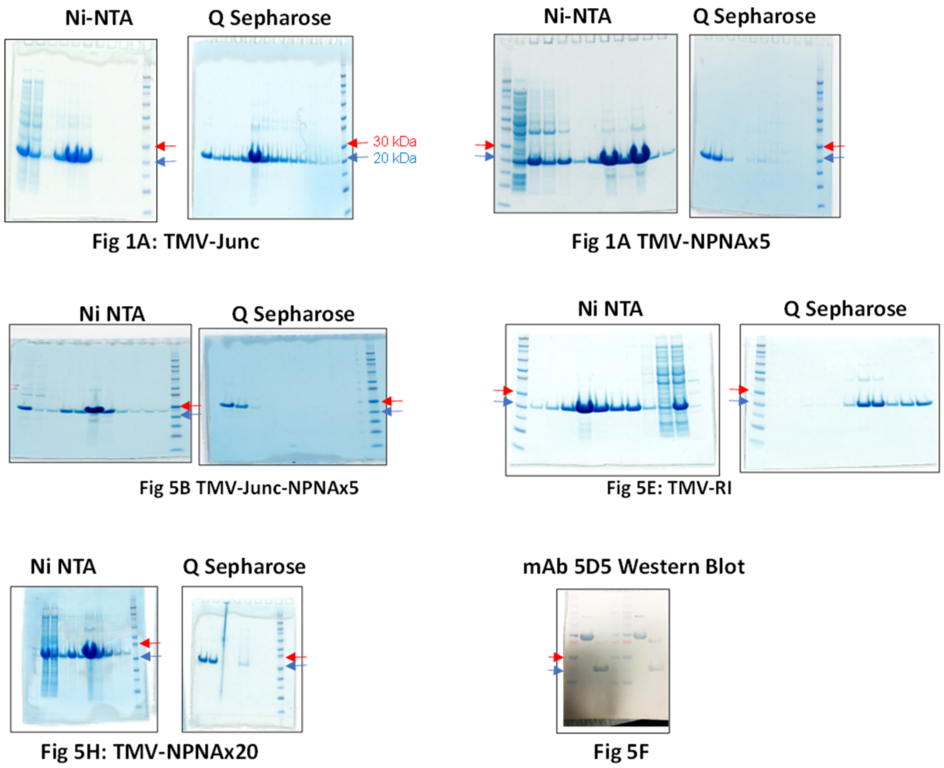

Supplementary Figure 4. Uncropped coomassie blue stained SDS-PAGE gels and western blots used to generate the corresponding figures for the manuscript (see Fig numbers). Novex Prestained Marker was used and 30 kDa (red) and 20 kDa (blue) marker bands are shown as arrows.
